# Peer-Assisted Learning in Undergraduate Medical Education for Resilience and Well-being

**DOI:** 10.1007/s40670-022-01702-x

**Published:** 2022-12-21

**Authors:** Cathy Snapp, Casey Bassett, Amy Baldwin, Janette R. Hill, Ruth DeBusk

**Affiliations:** 1 Office of Personalized Health and Well-being, Augusta University/ University of Georgia Medical Partnership University of Georgia Health Sciences Campus, Prince Avenue, Athens, GA 30602 USA; 2 Department of Pathology, Augusta University/University of Georgia Medical Partnership Athens, Augusta, GA USA; 3 Department of Biochemistry and Molecular Biology, Augusta University/University of Georgia Medical Partnership, Augusta, GA USA; 4 School of Social Work, University of Georgia, Augusta, GA USA; 5 Learning, Design, & Technology, University of Georgia, Augusta, GA USA; 6 MBN Systems, LLC, Tallahassee, USA

**Keywords:** Peer coaching, Peer-assisted learning, Undergraduate medical education (UME) resilience, Well-being emotional regulation

## Abstract

Peers are a powerful resource in the learning process. Peer-assisted learning (PAL) is a high value activity for the peer and the coach. We report here a PAL activity focused on resilience and well-being for first-year undergraduate medical students. The model and lessons learned are described.

Medical students and physicians experience a high rate of burnout, suicide, mental health, and substance use disorders, and peers provide support for assisting with these challenges through modalities such as peer-assisted learning (PAL) [1]. Numerous studies support the effectiveness of PAL models in medical education that (1) encourages a safe learning environment, influencing the depth of learning that takes place; (2) fosters the development of close relationships with peer-tutors with whom learners share a strong sense of shared mission; and (3) enables peer-coaches to feel equipped to understand their trainees’ wider sociocultural context [2]. Introducing PAL models of resilience education early in a trainee’s career likely provides well-being benefits such as inclusive peer relationships, skills of positive emotional regulation, and the use of lifestyle choices to build resilience, and has been shown to have the potential to enhance students’ self-perception of their knowledge base, preparing them for the rigorous academic environment of medical school [1]. The PAL experience provides a valuable foundation for medical learners to develop best practices for well-being. Participation will potentially prepare them to provide effective healthcare to their future patients.

During our students’ pre-clerkship training at the AU/UGA Medical Partnership, we use myriad ways of peer learning, including peer tutors. The cornerstone of our pre-clerkship curriculum is case-based learning, where our students assist their peers in both learning medical knowledge and practicing communication skills. Similarly, clinical skills, simulation, community health experience, and inter-professional learning events are facilitated within small group settings; end-of-course evaluations demonstrate students find these learning modalities effective and enjoyable.

We recently created a PAL session for orientation with incoming first-year students that focuses on positive emotional (PE) regulation to supplement the fostering of interactive, small group learning with well-being strategies that students can learn and practice from day 1 of medical school. This session included self-monitoring of heart rate variability (HRV) in the autonomic nervous system through environmental and psychological inputs. These biofeedback strategies use HRV with heart-focused breathing and positive emotion to increase resilience and well-being [3]. Prior to the PAL coaching session, peer coaches (year 2 and 3) were trained to use HRV and mindfulness strategies for emotional self-regulation to better coach their peers.

Students were introduced to the concepts of autonomic nervous system balance and HRV as they relate to resilience and overall well-being. Additionally, techniques for developing self-regulation, such as (1) how emotions influence the nervous systems, (2) PE and its influence on stress reduction, and (3) daily practices that promote greater resilience, were employed (introduced). Peer coaches led small breakout groups and provided real-life applications of emotional self-regulation strategies in what could be perceived as stressful situations (e.g., exams).

The goal of this training focused on increasing motivation to use PE regulation strategies by (a) selecting a learning situation that may be improved with PE, (b) modifying the learner’s perspective and subsequent response to that situation, (c) redirecting the learner’s attention towards specific features or details of situations that might increase PE, (d) changing the learner’s appraisal of an emotion-eliciting stimulus, and (e) experientially, physiologically, or behaviorally expressing the learner’s ongoing PE to build resilience. Sixty first-year students participated in the training. Pre- (*n* = 56) and post-surveys (*n* = 51), using open-ended and Likert questions, evaluated the learners’ perceptions of the session (1) goals, (2) expectations, (3) challenges, and (4) strategy use. The majority (75%, *n* = 38) of the post-survey respondents indicated their goals were met: strategies for emotional regulation, health promotion, and making connections with peers. Most respondents (88%, *n* = 44) indicated their expectations were met, particularly learning strategies for well-being, with some indicating exceeding expectations, while 82% (*n* = 42) reported their challenges were addressed, such as having a safe space to share and practice to techniques that can be integrated into busy schedules. Almost all respondents (96%, *n* = 49) shared how they anticipated using emotional regulation strategies during stressful situations and how to include the practices on a daily basis.

Our innovative approach for the goal of emotional self-regulation for developing resilience and well-being in first-year medical students involved peers sharing personal benefits and practical applications. Discussions promoted connections between and among coaches and learners, which helps initiate a supportive network that will serve all in the future. Peer coaches developed teaching experience that meets Accreditation Council for Graduate Medical Education competency requirements. Future training will address lifestyle choices to support greater well-being. Through PAL, participants are more likely to practice lifestyle-oriented care for themselves and their patients by promoting less reactivity, greater mental clarity, and better work-life balance.

## Data Availability

The datasets generated during, and/or analyzed during, the current study are available from the corresponding author on reasonable request.
